# Hemoglobin A1c and risk of left atrial thrombus and spontaneous echo contrast in non-valvular atrial fibrillation patients

**DOI:** 10.1186/s40001-017-0257-x

**Published:** 2017-04-21

**Authors:** Rong-Ren Kuang, Fang-Zhou Liu, Yun-Peng Li, Wei-Dong Lin, Hua-Sheng Liang, Ai-Hua Chen

**Affiliations:** 1 Department of Cardiology, Zhujiang Hospital, Southern Medical University, No. 253, Gong Ye Road, Guangzhou, 510282 China; 2 Department of Cardiovascular, Guangdong Cardiovascular Institute, Guangdong General Hospital, Guangdong Academy of Medical Sciences, Guangzhou, China; 3 Department of Cardiology, Longgang Central Hospital, Shenzhen, China

**Keywords:** Atrial fibrillation, Hemoglobin A1c, Left atrial thrombus, Spontaneous echo contrast, Prothrombotic state

## Abstract

**Objectives:**

To evaluate the relationship between hemoglobin A1c (HbA1c) and risk of left atrial thrombus/spontaneous echo contrast (LAT/SEC) in non-valvular atrial fibrillation (AF) patients.

**Methods:**

In this retrospective study, 1158 consecutive non-valvular AF patients undergoing transesophageal echocardiography prior to radiofrequency catheter ablation or electric cardioversion were enrolled. Baseline characteristics were collected and analyzed.

**Results:**

There were 87 (7.5%) patients with LAT/SEC. The HbA1c levels in the patients with LAT/SEC were significantly higher than that in patients without LAT/SEC (6.13 ± 0.41 vs. 5.89 ± 0.45 μmol/L, *P* < 0.001). The optimal cut-off point for HbA1c predicting LAT/SEC was 6.1% determined by receiver-operating characteristic curve. The area under the curve is 0.788 (95% confidence interval: 0.764–0.812). HbA1c ≥6.1% was an independent risk factor for LAT/SEC (odds ratio, 1.74; 95% confidence interval, 1.01–2.98; *P* = 0.045).

**Conclusions:**

Elevated HbA1c indicated a significantly increased risk for LAT/SEC in non-valvular AF patients. HbA1c might have significance in predicting the risk for prothrombotic state in non-valvular AF patients.

## Background

Atrial fibrillation (AF), known as the most common sustained form of rhythm disturbance, adversely affects the quality of life of millions of people [1]. AF is independently associated with a fivefold increased risk of stroke [2, 3]. Approximately 90% of cerebral thromboembolism generate from left atrium in non-valvular AF patients [4]. Left atrial thrombus (LAT) and spontaneous echo contrast (SEC) are known to be independent predictors of a higher incidence of embolic events [5, 6]. So LAT/SEC detected by transesophageal echocardiography (TEE) may be regarded as a pre-thromboembolic status.

Elevated hemoglobin A1c (HbA1c) is a well-known marker of chronic hyperglycemia. It is directly associated with stroke risk [7]. Elevated HbA1c is also associated with the risk of poor outcome and mortality in ischemic stroke patients with or without diabetes [8]. However, the relationship between HbA1c and LAT/SEC has not been established. The aim of the current study was to evaluate the relationship between HbA1c and risk of LAT/SEC in non-valvular AF patients with or without diabetes.

## Methods

### Study population

From July 2009 to May 2014, 1203 consecutive AF patients who underwent TEE prior to radiofrequency catheter ablation (RFCA) or electric cardioversion were screened in a retrospectively established database provided by Guangzhou Zhujiang Hospital and Guangdong cardiovascular institute. The patients with the following conditions were excluded: (1) structural heart disease, including congenital heart disease, rheumatic heart disease, valvular heart disease, infective endocarditis, history of cardiothoracic surgery, and cardiomyopathy; (2) factors influencing HbA1c, such as anemia, chronic renal failure, chronic liver disease, rheumatoid arthritis; (3) patients who refused to participate in the study. Finally, 1158 patients were included in the analyses. After admission, warfarin and antiplatelet medications were discontinued, and all the patients received subcutaneous low-molecular weight heparin (Enoxaparin, Sanofi-Aventis, France) twice per day as bridge therapy. Baseline characteristics were collected at admission, such as sex, age, and medical history (type of AF, heart failure, hypertension, diabetes mellitus, stroke/TIA, vascular disease, and so on). The patients underwent physical examinations, including blood pressure, electrocardiography, echocardiography, TEE, and blood testing (HbA1c, Uric acid, D-dimer, and low-density lipoprotein cholesterol). Patients were categorized into LAT/SEC group and non-LAT/SEC group by TEE results. The Ethics Study Committee at Guangdong General Hospital approved the study protocols, and informed consent was not necessary because of the observational nature of this study. The information of all of the patients was anonymized and de-identified prior to analysis.

### Blood samples

Blood samples for HbA1c were obtained on the first 24 h after admission. HbA1c was estimated using the high-performance liquid chromatography analysis on the D-10 Hemoglobin Testing System (Bio-Rad Laboratories, California, USA).

### TEE examination

All patients received local pharyngeal anesthesia (1% lidocaine spray) before TEE examination. The patient lay supine in the left lateral position when the transesophageal probe was introduced. Live images were interpreted by experienced physicians. LAT was diagnosed if a well-circumscribed echogenic mass detected in the appendage or body of the atrium appeared to be distinct from the underlying endocardium, was not caused by pectinate muscles, and was detected in >1 imaging plane. SEC was diagnosed if dynamic smog-like echoes appeared in the appendage or body of the atrium with a characteristic swirling motion distinct from a white noise artifact after properly adjusting the gain setting [9].

### CHADS_2_ score and CHA_2_DS_2_-VASc score

Stroke risk was then evaluated by CHADS_2_ score and CHA_2_DS_2_-VASc score [10]. The CHADS_2_ score was calculated as follows: 2 points were assigned for a history of stroke/TIA and 1 point was assigned for congestive heart failure, hypertension, age older than 75 years, and diabetes mellitus (DM). CHA_2_DS_2_-VASc score was calculated as follows: 2 points for a history of stroke or TIA, or age ≥75; and 1 point for age 65–74 years, history of hypertension, DM, recent cardiac failure, vascular disease, and female sex.

## Statistical analysis

Continuous variables are expressed as the mean ± SD, whereas categorical variables are expressed as numbers and proportions. Student *t* test or one-way ANOVA analysis was performed to compare the significance of differences in continuous or categorical variables. Mann–Whitney *U* test was performed when variables were not in normal distribution. To compare categorical variables, Chi square test or Fisher exact test was performed. The receiver-operating characteristic (ROC) curve analysis was performed to assess the efficiency of HbA1c in predicting LAT/SEC. The ROC curve was constructed by plotting sensitivity vs. 1-specificity. The area under the curve (AUC) and associated 95% CI were calculated. The optimal cut-off points for the variables predicting LAT/SEC were determined by the ROC curve. Multivariate logistic regression analysis was performed to identify the potential independent predictors for LAT/SEC. Results were considered significant for values of *P* < 0.05. All statistical analyses were carried out using IBM SPSS Statistics 19.0 (IBM Inc., NY, USA).

## Results

The baseline characteristics of the LAT/SEC group and the non-LAT/SEC group are shown in Table 1. The retrospective study included a total of 1158 patients (male 389, female 769, mean age of 56.84 ± 12.22). There were 87 (7.5%) patients with LAT/SEC. The patients with LAT/SEC were of older age, higher proportion of age ≥65 years, higher proportion of female, and had higher SUA level, larger left atrial diameter, lower ejection fraction, higher CHADS_2_ score, and higher CHA_2_DS_2_-VASc score than that in the patients without LAT/SEC. With regard to the medical history factors, the proportion of non-paroxysmal AF, heart failure, hypertension, type 2 diabetes mellitus, stroke/TIA, and vascular disease were significantly higher in the patients with LAT/SEC than that in the patients without LAT/SEC.

**Table 1:** Baseline characteristics of participants

Variables	LAT/SEC group (*n* = 87)	Non-LAT/SEC group (*n* = 1071)	*P*
Age (years)	61.47 ± 10.78	56.54 ± 12.30	<0.001*
Female, *n* (%)	38 (43.68)	351 (32.77)	0.045*
Non-paroxysmal AF, *n* (%)	38 (43.68)	175 (16.34)	<0.001*
Type 2 diabetes mellitus, *n* (%)	31 (36.63)	148 (13.82)	<0.001*
Hypertension, *n* (%)	61 (70.11)	455 (42.48)	<0.001*
Stroke/TIA, *n* (%)	9 (10.34%)	19 (1.77)	<0.001*
Heart failure, *n* (%)	27 (31.03)	129 (12.04)	<0.001*
Vascular disease, *N* (%)	17 (19.54)	71 (6.63)	<0.001*
COPD, *N* (%)	21 (18.27)	211 (19.70)	0.855
Age ≥65, *N* (%)	38 (43.68)	310 (28.94)	0.005*
Age ≥75, *N* (%)	6 (6.90)	46 (4.30)	0.275
LVDd, (mm)	46.79 ± 4.67	45.84 ± 4.54	0.070
LAd, (mm)	41.36 ± 5.58	36.29 ± 5.66	<0.001*
LVEF, (%)	63.86 ± 6.89	65.79 ± 7.02	0.014*
Uric acid, (μmol/L)	395.98 ± 99.68	356.49 ± 78.04	<0.001*
Hb1Ac, (%)	6.13 ± 0.41	5.89 ± 0.45	<0.001*
LDL-C, (μmol/L)	3.06 ± 0.93	2.90 ± 0.81	0.336
D-dimer, (μg/L)	220.45 ± 210.42	247.87 ± 439.71	0.412
CHADS_2_ score	1.64 ± 1.27	0.76 ± 0.66	<0.001*
CHA_2_DS_2_-VASc score	2.71 ± 1.68	1.45 ± 1.30	<0.001*

*AF* atrial fibrillation, *TIA*: transient ischemic attack, *LVDd* left ventricular diastolic dimension, *LAd* left atrial dimension, *LVEF* left ventricular ejection fraction, *LDL* low-density lipoprotein, *COPD* chronic obstructive pulmonary disease

* With statistical significance

**Fig. 1:**
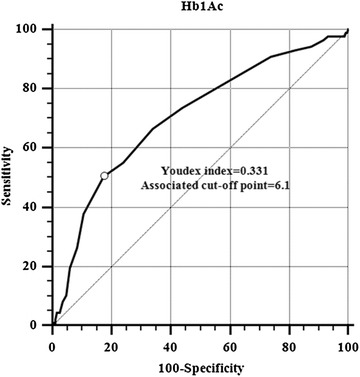
The receiver-operating characteristic (ROC) curve for hemoglobin for predicting LAT/SEC. The area under the ROC curve = 0.788 (95% confidence interval: 0.764 to 0.812)

The HbA1c level in the patients with LAT/SEC were significantly higher than that in the patients without LAT/SEC (6.13 ± 0.41 vs. 5.89 ± 0.45 μmol/L, *P* < 0.001). The optimal cut-off point for HbA1c predicting LAT/SEC was 6.1% determined by ROC curve (Fig. 1). The AUC is 0.788, which indicated HbA1c had the moderate predictive value.

We used multivariate logistic regression analysis to identify the relationship between the potential risk factors and LAT/SEC (Table 2). After adjustment for female, non-paroxysmal AF, type 2 diabetes mellitus, hypertension, stroke/TIA, heart failure, vascular disease, LA dimension ≥40 mm, and age, HbA1c ≥6.1% was an independent risk factor for LAT/SEC (odds ratio, 1.74; 95% confidence interval, 1.01–2.98; *P* = 0.045).

**Table 2:** Multivariate logistic regression analysis for LAT/SEC

Variables	Odds ratio	95% CI	*P*
Female	1.91	1.16–3.17	0.012*
Non-paroxysmal AF	3.46	2.07–5.78	<0.001*
Type 2 diabetes mellitus	1.72	0.97–3.12	0.057
Hypertension	1.74	1.00–3.04	0.050*
Stroke/TIA	3.32	1.23–9.01	0.018*
Heart failure	2.01	1.16–3.48	0.013*
Vascular disease	2.04	1.02–4.07	0.043*
Age ≥65 years	1.30	0.76–2.23	0.339
Age ≥75 years	0.65	0.22–1.95	0.446
LA dimension ≥40 mm	4.04	2.46–6.64	<0.001*
Hb1Ac ≥6.1%	1.74	1.01–2.98	0.045*

*AF* atrial fibrillation, *TIA* transient ischemic attack, *LA* left atrial

* With statistical significance

## Discussion

In this study we investigated the relationship between HbA1c and risk of LAT/SEC in a total of 1158 non-valvular AF patients of southern China with or without diabetes. We found HbA1c levels were significantly higher in LAT/SEC group; HbA1c ≥6.1% was an independent risk factor for LAT/SEC in AF patients; and it was a moderate predictive value for LAT/SEC. So HbA1c might have significance in predicting the risk for prothrombotic state in non-valvular AF patients.

Radiofrequency catheter ablation are often used to restore sinus rhythm in AF patients. It is associated with an increased risk for stroke. TEE is the most sensitive and specific technique to detect left atrium/left atrial appendage (LA/LAA) thrombi as a potential source of thromboembolic events such as ischemic stroke. TEE can also detect features associated with an increased risk of LA/LAA thrombus formation, including reduced LAA flow velocity and SEC [11]. So the use of TEE to guide the management of AF is a validated strategy for AF patients before RFCA. At present, 2 stroke risk stratification schemes have been widely used in clinical practice, including the CHADS_2_ score and the CHA_2_DS_2_-VASc score. However, these 2 schemes have only modest predictive value for “high-risk” patients, with a c-statistic of approximately 0.6 [12]. Thus, many potential risk factors need to be assessed.

HbA1c is a kind of β-*N*-(1-deoxy)-fructosyl hemoglobin contained within the red blood cell which is glycated in varying amounts depending on blood glucose levels over time. This protein can be found in the red blood cell for its entire life span of approximately 120 days [13], so HbA1c levels could reflect the mean glucose range for the previous 2–3 months in patients with or without diabetes mellitus. HbA1c testing does not require patients to fast and its intra individual variability is small, so it is a good value for hyperglycemic and diabetic diagnosis and monitoring. Moreover, HbA1c testing also can find pre-diabetic status. Recent studies have shown that HbA1c is related to the onset of AF. Dublin et al. found diabetes was associated with higher risk of developing AF, and risk was higher with worse glycemic control defined by higher HbA1c [14]. Sandhu et al. also found increasing HbA1c levels were preferentially associated with the early development of non-paroxysmal AF in women without AF or cardiovascular disease (CVD) at baseline [15]. Another study showed that the level of HbA1c, especially in patients with HbA1c >6.5%, was associated with AF occurrence [16]. The precise pathophysiologic mechanisms of how AF is associated with HbA1c are not yet well known. The proposed mechanisms may include autonomic remodeling, structural remodeling, electrical remodeling, electromechanical remodeling, connexin remodeling, and oxidative stress [17].

Many studies demonstrated that HbA1c is associated with thromboembolic events. Ikeda et al. reported that elevated HbA1c levels even within non-diabetic level are an independent risk factor for CVD, especially coronary heart disease and ischaemic stroke. With regard to CVD subtype, the positive associations between HbA1c levels and the risk of ischaemic stroke were significant [18]. Oh et al. also found higher HbA1c indicated a significantly increased risk for ischemic stroke after adjusting for other confounding variables in non-diabetic Korean adult males [19]. Saliba et al. demonstrated Glycated hemoglobin is directly associated with stroke risk, and it improves the predictive accuracy for stroke in diabetic patients with AF [7]. However, the relationship between HbA1c and LAT/SEC remains unknown. In the present study, we found that elevated HbA1c level is associated with increased risk for LAT/SEC in non-valvular AF patients, so it could be a predictor of LAT/SEC in patients with non-valvular AF.

Hyperglycemia plays an important role in the development of many abnormalities including endothelial dysfunction, platelets hyperreactivity, increased coagulability, and fibrinolytic impairment [20]. Glycosylation is a non-enzymatic reaction induced by chronic hyperglycaemia. HbA1c is a precursor of advanced glycation end products (AGEs) known as one of glycosylation’s products. AGEs elicit oxidative stress, inflammatory reactions, and thrombosis, thereby being involved in vascular damage [21, 22]. Hence, elevated HbA1c levels promote LAT/SEC in non-valvular AF patients.

Although this was not a prospective cohort study, we hypothesize that LAT/SEC in non-valvular AF patients may be influenced by HbA1c level. In order to prevent the LAT/SEC, a regimen to lower HbA1c, such as nutrition control and physical exercise and glucose control by drug therapy may be recommended.

Several limitations have been identified in the present study. Firstly, the present study is a retrospective study, which needs more research in the future. Secondly, the exact mechanism of HbA1c levels linking to LAT/SEC has not been well explored. Thirdly, the population in this study might not reflect the general AF population, because the subjects in this study were from southern China. Finally, considering the possible gender differences, we also failed to show the usefulness of HbA1c as a predictor of LAT/SEC with different genders.

## Conclusion

Elevated HbA1c level is associated with increased risk for LAT/SEC in non-valvular AF patients. HbA1c might have significance in predicting the risk for prothrombotic state in non-valvular AF patients. Control of glycemic metabolism may play an important role in reducing the risk for LAT/SEC, especially for subjects with HbA1c ≥6.1%.
